# Proteomic analysis of aqueous humor from patients with primary open angle glaucoma

**Published:** 2010-12-18

**Authors:** Xiaoming Duan, Peng Xue, Ningli Wang, Zhe Dong, Qingjun Lu, Fuquan Yang

**Affiliations:** 1 Beijing Tongren Eye Center, Beijing Tongren Hospital, Capital Medical University, Beijing Ophthalmology & Visual Sciences Key Laboratory, Beijing, China; 2 Laborotary of Proteomics, Institute of Biophysics, Chinese Academy of Sciences, Beijing, China; 3 Beijing Tongren Eye Center, Beijing Tongren Hospital, Beijing Institute of Ophthalmology, Capital Medical University, Beijing Ophthalmology & Visual Sciences Key Laboratory, Beijing, China

## Abstract

**Purpose:**

Primary open angle glaucoma (POAG) is a leading cause of irreversible blindness on a global level. Researchers have yet to specify the exact mechanisms of POAG; the respective relationships between POAG and elevated intraocular pressure (IOP), as well as optic neuropathy, remain particularly unclear. It is known, however, that the expression profile for some proteins in the aqueous humor (AH) changes in some diseases, and that AH changes play important roles in elevated IOP. To identify the possible roles of these AH proteins in POAG, a proteomic analysis of the AH compositions of POAG patients’ eyes was performed and compared with those derived from paired, non-POAG cataract (control) eyes.

**Methods:**

We used Bradford’s method to determine total protein concentration in AH, and analyzed separation profiles via two-dimensional (2D) gel electrophoresis. We used silver stain to determine gel proteins, and analyzed separation profiles to assess spot density differences between POAG and non-POAG patients. These gel spots were isolated and identified via mass spectrometry. Prostaglandin H2 D-isomerase (PGDS) in AH were analyzed by western Blotting.

**Results:**

There was no significant difference between the total protein concentration in AH of POAG patients and that in AH of non-POAG patients. A total of seven spots were increased in 2D gels from POAG patients. The spots were derived from PGDS, caspase 14 precursor, transthyretin, cystain C, albumin precursor, and tranferrin. And PGDS in AH from patients was more than from controls.

**Conclusions:**

The protein composition in AH was significantly different in POAG patients versus non-POAG patients. The identified proteins could be a potential biomarker for POAG and may play a role in the mechanisms of elevated IOP and optic neuropathy in POAG.

## Introduction

Glaucoma is a leading cause of irreversible visual impairment and blindness on a global level [1]. Primary open angle glaucoma (POAG), a common type of glaucoma, is progressive optic neuropathy characterized by a distinct pattern of optic nerve damage and visual field loss. In this type of neuropathy elevated intraocular pressure (IOP) due to increased resistance of AH outflow through the trabecular meshwork (TM) is one of the most significant risks. However, the pathogenesis of POAG, especially elevated IOP, is not well understood.

AH is an important intraocular fluid responsible for the supply of nutrients to and removal of metabolic wastes from the avascular tissues of the eye [2]. It contains proteins secreted from anterior segment tissues [3]. It is known that protein levels in AH vary and change in many eye diseases [4-8]. Some protein changes in AH correlate with the mechanisms or prognoses of many eye disorders [9-13]. Previous studies have focused on investigating changes in AH of POAG patients. It was reported that transforming growth factor beta 2 (TGFβ2) and plasminogen activator inhibitor-1 (PAI-1) are elevated in patients with POAG [12,14,15] and TGFß2 could play roles in POAG through the TGFß receptor mediated signal pathway [10]. Such findings suggest that some proteins in AH might be involved in the development of POAG.

In some previous studies, ELISA was used to examine the cytokines or some growth factors [7,13]. However, due to the little sampling volume and low concentration proteins in AH, a wide range of the protein studies was limited via traditional methods. Proteomics has expanded the opportunities to discover disease-specific proteins involved in AH circulation, and has been used to study eye diseases such as acute corneal rejection [16] and myopia [17]. The proteomic techniques used include protein separation by two-dimensional gel electrophoresis (2-DE) and characterization by mass spectrometry of peptides, amino acid sequencing and bioinformatics analysis. High resolution two-dimensional (2D) PAGE (PAGE) is a technique for analyzing several hundred proteins in tissues, fluids or cells using only a few microliters of sample, and is therefore theoretically ideal for analyzing limited volumes of AH.

In this study, we investigated the differential proteomes in five POAG patients (patients) as well as in matched, non-POAG cataract patients (controls). Abnormal expressions and distributions of proteins from AH were identified and evaluated in age-paired clinical specimens. It is possible to achieve a better understanding of the molecular events involved in POAG development, and generate essential data needed for elaborating more effective strategies designed to help identify new biomarkers and/or treatments.

## Methods

### Sample collection

We collected AH samples at the Beijing Tongren Eye Center, Capital Medical University, China. We obtained an informed consent from each patient. During the period from October 2005 to May 2006, we collected AH samples from five patients with POAG (patients) and from five cataract patients without POAG (controls).

The glaucoma patients had uncontrolled IOP despite the use of well tolerated medical therapy. They experienced moderate or severe visual field defects and optic disc cupping. We found that the control cases had normal IOP (less than 20 mmHg in at least two measurements separated by more than one day) and were undergoing routine senile cataract surgery for visual rehabilitation. In addition, the glaucoma patients and the control cases were age- and gender-matched. Case exemptions included patients with other ophthalmic diseases, systemic diseases (i.e., diabetes mellitus, arthritis), and patients who had undergone laser or intraocular surgery.

The study followed the tenets of the Declaration of Helsinki, and informed written consent was obtained from all patients and controls after we explained the nature and possible consequences of the study. The study protocol was approved by the Medical Ethical Committee of Beijing Tongren Hospital, Capital Medical University, Beijing.

All AH samples were obtained before surgery. Approximately 100~200 μl AH was collected by a 26 gauge ophthalmic cannula under a binocular microscope. The AH was immediately stored in aliquots of 50 μl in 1.5 ml microtubes at −80 °C until further analysis.

### Total protein quantization

Total AH protein concentration was determined according to the Braford method (Bio-Rad Laboratories, Hercules, CA) following the manufacture’s protocol as dictated in a previous study [17].

### Two-dimensional gel electrophoresis

The principle procedure for running 2D gels has been described in detail in our previous study [17]. We solubilized 45 ml of the AH samples in 55 μl lysis solution and 100 μl rehydration solution, to achieve a total volume of AH, lysis solution, and rehydration solution resulting in 200 μl. We used the final solution to rehydrate immobilized pH gradient stripes overnight with linear pH 3–10 at a length of 110 mm (Immobilize Drystripes; Amersham Biosciences Inc., Amersham, UK). For the first dimension, we performed isoelectric focusing on an Ettan Multiphor flat bed unit with minor modifications of the manufacturer’s instructions (Amersham Biosciences Inc., Amersham, UK). For the second dimension, we separated samples via PAGE on a separating gel (1 mm thickness, 12% SDS gel; Hoefer SE600 Ruby, Amersham Biosciences Inc.). We applied a 20 mA constant current per gel, and ran the gel until the dye reached the approximate bottom.

The separated proteins within the gels were fixed over a 2 h period in a 40% ethanol, 10% acetic acid solution. We then silver stained the gel proteins according to manufacturer protocol (Silver stain plus kit; BioRad).

### Analysis of two-dimensional gels

The stained gels were scanned with an Imagescanner (Amersham Biosciences Inc.) and analyzed with Imagemaster 2D platinum software (Amersham Biosciences Inc.). We matched the gels according to the number of user-defined landmarks on each gel. Each spot was quantified according to the % volume of the stained spot. We chose to work only with spots that showed greater than two ratios in % volume between patients and controls. The differences in the spot % volumes were compared with Mann–Whitney test.

### Protein Identification by liquid chromatography-mass spectrometry (LC MS/MS)

The principle procedure for protein identification has been described in detail in our previous study [17]. We proteolyzed the gel pieces with 20 ng of modified trypsin (Promega Biotech Co., Ltd, Madison, WI) in 25 mM ammonium bicarbonate (AmBic) overnight at 37 °C. We then collected supernatant, and further extracted peptides in 0.1% formic acid, 60% acetonitrile. Peptide extracts were vacuum-dried and resuspended in 20 ml of 0.1% formic acid solution for mass analysis. We performed a LC-MS/MS analysis in a linear trap quadrupole (LTQ;  (ThermoFinnigan, San Jose, CA) coupled online with a nano-liquid chromatography (Nano LC) system. We searched all tandem spectra against a *H. sapiens* NCBI reference database (human.ncbi) using SEQUEST (version 2.7). Results were filtered by Xcorr (the cross-correlation value from the search) +1>1.9,+2>2.5,+3>3.75, sp>500, *Δcn* (the Delta Correlation value) >0.1, Rsp<=5. Every protein identified matched at least two peptides.

### Prostaglandin H2 D-isomerase (PGDS) detection using western blotting

The existence of PGDS proteins in aqueous humor of glaucoma patients was confirmed using western blotting. AH was separated on 10% SDS–PAGE and transferred onto a 0.2 μm polyvinylidene fluoride membrane (Millipore, Molsheim, France) at 220 mA for 2 h in a Bio-Rad Transblot electrophoretic transfer cell (Bio-Rad). The non-specific reaction were blocked with 5% non-fat milk in TBS, the membrane was washed in TBS and incubated with anti-PGDS antibody (0.3 μg/ml), followed by three washing with TBST and incubation with horseradish peroxidase-conjugated mouse anti-rabbit immunoglobulin (1:4,000; DakoCytomation, Glostrup, Denmark). The PGDS-anti- PGDS complex was chemoluminescence was recorded on Fuju X-ray film (Fuji Photo Film Co. Ltd, Tokyo, Japan).

### Statistics

We used SPSS Version 11.0 software (SPSS Inc, Chicago, IL) to calculate p-values achieved using the Mann–Whitney test for all data obtained. A p<0.05 was required for the results to be considered statistically significant.

## Results

A total of 10 AH samples were included in this study, five from POAG patients (mean age 43.60±12.58 years, three males and two females) and five from age- and gender-matched non-POAG cataract patients (mean age 49.80±5.89 years, three males and two females). There ware no statistically significant differences between the two groups with regard to age (p=0.55). Clinical data from the 10 patients are summarized in Table 1.

**Table 1:** Data from POAG patients and controls.

**Group**	**Number**	**Age**	**Sex**	**IOP (mmHg)**	**C/D ratio**
Patient	1	51	F	18	0.8
	2	32	M	25	0.6
	3	55	F	18.5	0.6
	4	67	M	16	0.7
	5	52	M	21	0.5
Control	1	52	F	13.8	0.3
	2	40	M	12.4	0.1
	3	53	F	10.9	0.2
	4	68	M	12.9	0.3
	5	49	M	16.7	0.1

### Protein content in AH from patients and controls

The mean total protein level in AH from POAG patients was 0.4382±0.1675 mg/ml (Range=0.2796~0.6517 mg/ml) while that from control patients was 0.3124±0.0567 mg/ml (range=0.2484~0.3984 mg/ml; Table 2). Although the difference between POAG patients and control patients was not statistically significant (p=0.31) total protein levels in POAG patients were greater than that of control patients.

**Table 2:** Measurement of total protein in AH from patients and controls.

**Group**	**Total protein (mg/ml)**	**p-value**
Patients	0.4382±0.1675	0.31
Controls	0.3124±0.0567	

### Two-dimensional gel electrophoresis patterns

**Figure 1:**
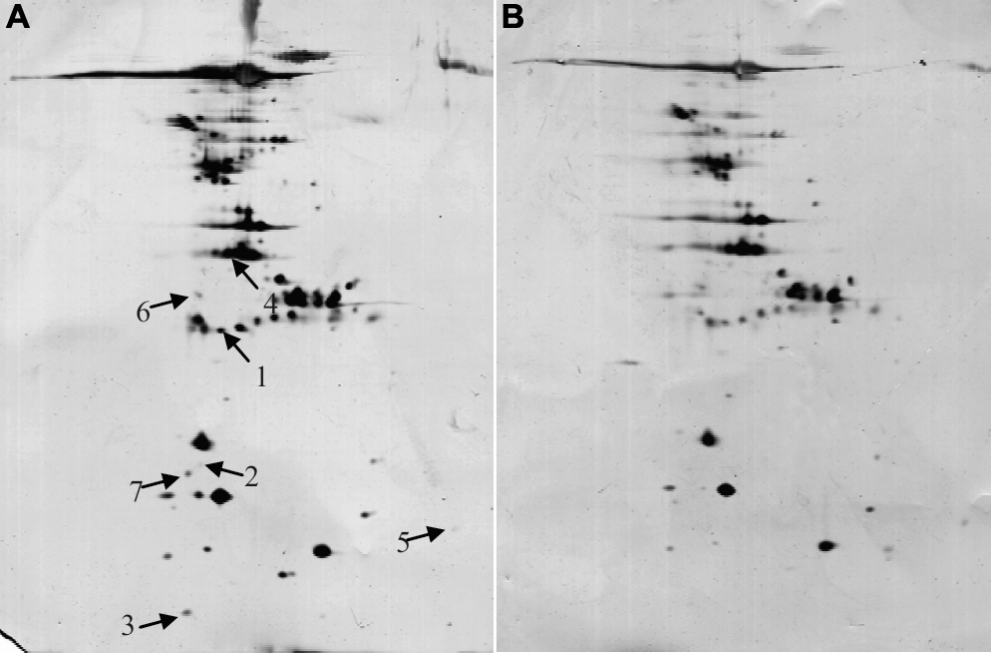
Silver-stained 2D gels of AH.**A** and **B** show representative gels from a patient (**A**: patient number 1 in Table1) and a gel from a control subject  (**B**: control number 1 in Table1). Total protein concentration in AH was 0.3217 mg/ml from the patient and 0.2924 mg/ml from the control. Arrows and numbers show 8 spots in the patient sample with volumes significantly increased by values greater than 2 fold in the patients. The identities of the spots were derived from prostaglandin H2 D-isomerase (PGDS; spot 1), caspase 14 precursor (spot 2 and4), transthyretin (spot 3 and 5), cystain C (spot 6), albumin precursor (spot 7), and tranferrin (spot 8).

Figure 1 shows a representative gel from a patient (Figure 1A; patient number 1 in Table 1) and a gel from a control subject (Figure 1B; control number 1 in Table 1). Gels from POAG patients (Figure 1A) displayed more spots and more intensely silver stained spots than in gels from control patients (Figure 1B). There were significant differences in relative spot volumes (% volume) between POAG patients and control patients. The gel patterns from POAG patients showed greater % volumes than those of control patients (Table 3). No protein spots were found to be downregulated in POAG patients as compared with control patients.

**Table 3:** Results from mass spectrometry of seven spots identified by 2D gels to occur in AH from POAG patients (n=5) in an amount at least twice that observed in controls (n=5).

**Spot number**	**Protein description**	**Identified peptide**	***Δcn***	**Xcorr**	**MH^+^**	Ratio*	P-value**
1	Prostaglandin H2 D-isomerase	R.TMLLQPAGSLGSYSYR.S	0.55	5.69	1744.99	13.10	0.02
		K.AQGFTEDTIVFLPQTDK.C	0.52	5.19	1911.10		
		R.TM@LLQPAGSLGSYSYR.S	0.50	5.02	1760.99		
2	caspase 14 precursor	K.GHILELLTEVTR.R	0.47	4.04	1381.60	5.94	0.03
		R.DPTAEQFQEELEK.F	0.46	3.95	1564.63		
		K.RDPTAEQFQEELEK.F	0.26	3.78	1720.82		
		K.AREGSEEDLDALEHMFR.Q	0.43	4.35	2006.14		
		R.DPTAEQFQEELEKFQQAIDSR.E	0.48	4.31	2510.65		
3	transthyretin	R.GSPAINVAVHVFR.K	0.50	2.85	1367.58	2.18	0.04
		K.TSESGELHGLTTEEEFVEGIYK.V	0.57	4.51	2456.60		
		R.YTIAALLSPYSYSTTAVVTNPK.E	0.58	4.83	2361.68		
		R.YTIAALLSPYSYSTTAVVTNPKE.	0.56	5.15	2490.79		
		K.AADDTWEPFASGK.T	0.60	3.53	1395.46		
4	Albumin precursor	K.LVNEVTEFAK.T	0.54	3.65	1150.31	5.98	0.03
		R.FKDLGEENFK.A	0.27	2.66	1227.35		
		K.LVNEVTEFAK.T	0.57	3.57	1150.31		
5	Cystain C	R.ALDFAVGEYNK.A	0.42	3.51	1227.35	5.17	0.04
		R.LVGGPMDASVEEEGVR.R	0.44	4.02	1645.82		
		R.LVGGPM*DASVEEEGVR.R	0.49	3.46	1661.81		
6	Albumin precursor	K.YICENQDSISSK.L	0.44	3.51	1444.55	11.09	0.03
		K.VPQVSTPTLVEVSR.N	0.5	3.23	1512.73		
7	transferrin	R.FDEFFSEGCAPGSK.K	0.37	2.57	1578.68	5.06	0.03
		K.IECVSAETTEDCIAK.I	0.63	3.90	1726.91		

In the Table, M@ indicates methionine oxidation, adding 16.00 Da. The asterisk indicates the ratio between spot % volume of the five patients and the five controls. The double asterisk indicates spot % volumes compared between patients and controls with a Mann–Whitney test.

### Identification of proteins

**Figure 2:**
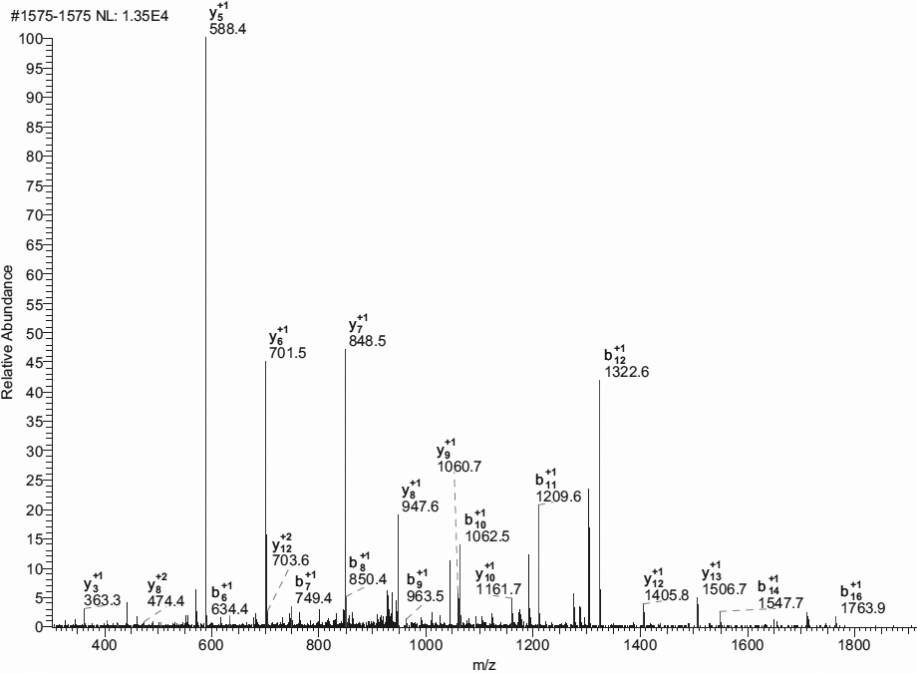
MS/MS spectrum of prostaglandin H2 D isomerase(PGDS). The protein peptide AQGFTEDTIVFLPQTDK ([M+H]2+=1911.10) was identified using this spectrum, which showed many characteristic y and b series ions.

Based on the above results, we isolated the seven protein spots for further analysis. Each spot was acquired from the gel and digested extensively with trypsin. The resulting peptides were applied to a nanoLC MS/MS for identification. Three peptides were captured and measured in spot 1, showing a significant protein increase in POAG patients. Figure 2 shows a typical MS/MS spectrum of its parent ion m/z=1911.10 (sequence: AQGFTEDTIVFLPQTDK). Many “y” and “b” series ions from this peptide were clearly identified in this spectrum. Sequence searching indicated that this peptide was from human prostaglandin H2 D-isomerase (PGDS). The remaining two peptides identified from protein spot 1 were also located in PGDS. The total sequence coverage was 17.40%.

Using the same methods, protein spot 2 showed a significant peptide increase in POAG patients, and was identified as caspase 14 with a sequence coverage of 21.10%. Significantly increased protein spots 3, 4, 5, 6, and 7 were identified as transthyretin, albumin, Cystain C, albumin, and transferrin, with sequence coverages of 48.30%, 3.30%, 18.50%, 4.27%, and 4.20% respectively.

### Western blotting analysis

**Figure 3:**
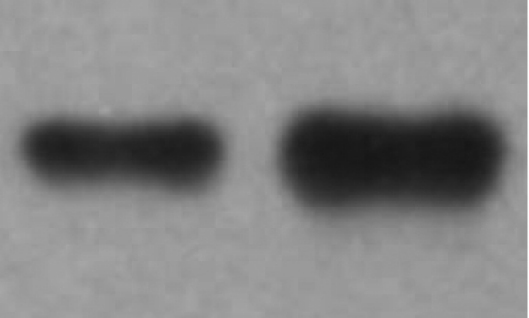
Western blotting analysis of PGDS in AH. The left lane is a sample from a control and the right lane is a sample from a POAG patient.

Anti-PGDS antibody-positive protein was present in AH. Immunoreactive protein strip of AH from POAG patients was more than that from controls. It suggested that PGDS in AH from POAG patients be up-regulated compared with that from controls (Figure 3).

## Discussion

Although POAG has been widely considered a multifactorial disease, IOP is the most important known risk factor for the development of glaucomatous optic nerve damage [18]. Reducing IOP can still be considered the most beneficial treatment in terms of halting visual field damage progression, even in normal pressure glaucoma. However, the mechanisms of elevated IOP have not been fully elucidated. At present, it is well known that elevated IOP could result from a variety of cell deaths in the trabecular meshwork [19]. AH, the circulated intraocular fluid, includes many kinds of cytokines, which could likely play significant roles in the trabecular meshwork.

Most recent insights regarding POAG have come from studies on animal models. Other studies of AH typically focus only on proteins. Grus et al. [20] has studied transthyretin changes in AH of POAG using surface enhanced laser desorption/ionization-time of flight-mass spectrometry (SELDI-TOF-MS) ProteinChip arrays and two-dimensional electrophoresis. In the study, it was known that the protein in AH was very low and that protein amount is affected by many factors, such as age, gender, and so on. If AH samples were pooled for analysis, it is possible that some subtle protein changes would be neglected. Therefore, in this study, we directed our attention to proteins in the AH collected directly from patients and analyzed AH protein changes strictly between POAG patients and paired non-POAG control patients. Such information might offer new insights to reveal the mechanism of POAG and identify potential biomarkers of this condition.

In this study, we compared AH proteins between POAG patients and non-POAG patients (as controls). The patterns of 2D electrophoresis gels in POAG patients were different from those of control patients, which indicated that the protein content in the AH changes with POAG development. However, the origin and function of AH proteins is still unknown. Results from previous studies have implicated that AH proteins could activate signaling cascades, which subsequently regulate cellular functions including mitosis, differentiation, motility and apoptosis. Such proteins play a vital role in corneal wound healing, mediating the proliferation of epithelial and stromal tissue and affect remodeling of the extracellular matrix (ECM).

In this study, we suggest that alterations in proteins could possibly contribute to the pathologic changes and complications of POAG in two ways - by directly affecting trabecular meshwork or as a response to elevated IOP.

First, we observed a significant increase of PGDS in our POAG patients and proved that by western blotting analysis. At present, the function of PGDS in ocular tissues is not clear. PGDS is responsible for the biosynthesis of prostaglandin D2 in the central nervous system and the genital organs and is secreted into the cerebrospinal fluid and the seminal plasma as beta-trace. It is a bifunctional protein that acts as both a retinoid transporter and a prostaglandin D2-producing enzyme [21]. Elevated PGDS levels have been observed in the serum of patients with renal impairment, diabetes mellitus, and hypertension. Recently, we demonstrated the ability of PGDS to induce apoptosis in a variety of cell types including epithelial cells, neuronal cells, and vascular smooth muscle cells [22,23]. Therefore, we suggest that PGDS might mediate the apoptosis of trabecular meshwork.

Second, there were few reports about caspase 14 in eyes. Caspase-14 is a unique member of the evolutionarily conserved family of cysteinyl aspartate-specific proteinases, which are mainly involved in inflammation and apoptosis [24]. Previous studies demonstrated that caspase 8 and caspase 9 are the main kinds of caspase that play roles in the apoptosis of ocular tissues. According to this study, caspase 14 might be involved in the apoptosis of ocular tissues by either directly mediating or inducing caspase 8 and caspase 9 activation.

In this study, we also observed a significant increase of transthyretin (TTR) and cystain C in our POAG patients. It was similar to the changes in cerebrospinal fluid (CSF) of Alzheimer disease (AD). It suggested POAG has similar mechanisms as AD, which is a progressive, neurodegenerative disease [20,25]. In addition, Grus et al. [20] reported that transthyretin was highly abundant in the AH of glaucoma patients, similar to our results.

In addition, we observed elevated transferrin in the AH of POAG. We suggest it was a response to elevated IOP. Transferrin was related to the point of inflammation [26], which usually occurs due to high IOP [27].

In our experiments, we identified additional spots, which might be derived from albumin, according to the characterization and a previous report [16]. However, the albumin fragments we obtained were different in pI and/or molecular weight. While we do not have a complete explanation for this finding, we speculate that it could be due to the degradation of albumin pathways in the AH our POAG patients.

In addition, the mean total protein level in AH from POAG patients (0.4382 mg/ml) was greater than that of non-POAG control patients. However, there was no significant statistical difference. We suggest that it is due to high SD in the POAG patients.

In conclusion, the results of our study revealed that the proteomic composition of AH was significantly different between POAG and control conditions. The proteins identified could play roles in the apoptosis of trabecular meshwork and serve as potential biomarkers for POAG development.
